# Simultaneous assessment of lung morphology and respiratory motion in retrospectively gated in-vivo microCT of free breathing anesthetized mice

**DOI:** 10.1038/s41598-022-17335-4

**Published:** 2022-08-02

**Authors:** Christian Dullin, Angelika Svetlove, Jana Zschüntzsch, Frauke Alves

**Affiliations:** 1 Institute for Diagnostic and Interventional Radiology, University Medical Center Goettingen, Goettingen, Germany; 2 Max-Plank-Institute for Multidisciplinary Sciences, Translational Molecular Imaging, Goettingen, Germany; 3 Institute for Diagnostic and Interventional Radiology, University Hospital Heidelberg, Heidelberg, Germany; 4 Clinic for Neurology, University Medical Center Goettingen, Göettingen, Germany; 5 Clinic for Haematology and Medical Oncology, University Medical Center Goettingen, Goettingen, Germany; 6 Cluster of Excellence ”Multiscale Bioimaging: from Molecular Machines to Networks of Excitable Cells” (MBExC), University of Goettingen, Goettingen, Germany

**Keywords:** Respiration, Preclinical research, Imaging techniques

## Abstract

Retrospective gating (RG) is a well established technique in preclinical computed tomography (CT) to assess 3D morphology of the lung. In RG additional angular projections are recorded typically by performing multiple rotations. Consequently, the projections are sorted according to the expansion state of the chest and those sets are then reconstructed separately. Thus, the breathing motion artefacts are suppressed at a cost of strongly elevated X-ray dose levels. Here we propose to use the entire raw data to assess respiratory motion in addition to retrospectively gated 3D reconstruction that visualize anatomical structures of the lung. Using this RG based X-ray respiratory motion measurement approach, which will be referred to as RG based X-ray lung function measurement (rgXLF) on the example of the *mdx* mouse model of Duchenne muscle dystrophy (mdx) we accurately obtained both the 3D anatomical morphology of the lung and the thoracic bones as well as functional temporal parameters of the lung. Thus, rgXLF will remove the necessity for separate acquisition procedures by being able to reproduce comparable results to the previously established planar X-ray based lung function measurement approach in a single low dose CT scan.

## Introduction

Lung diseases continue to present a large burden to public health, especially in industrialized countries. This situation might even worsen in the future due to the current coronavirus (COVID-19) pandemic. For a better understanding of the underlying pathomechanisms in lung related diseases as well as for testing the efficacy of novel therapies, preclinical studies in animal models are required. These models are typically realized in mice, which due to their small size and rapid breathing rate render in-vivo imaging very challenging. Non-invasive readouts, however, are a major asset in preclinical studies as they allow monitoring disease progression and/or response to therapy in the same animal over time. However, in lung diseases these studies are further hindered by complexity of the diseases that cause changes on anatomical, functional and cellular levels. Thus, a combination of different methods and imaging techniques is required for a comprehensive analysis^1^.

Computed tomography (CT) is the method of choice for lung imaging because air inside the lung acts as a natural negative contrast agent, allowing a detailed three dimensional (3D) anatomical characterisation. However, the working principle of CT, reconstructing cross-sections of the specimen by analyzing angular distributed projection images, requires the object/subject/mouse to remain static during data acquisition. Thus, breathing motion—if uncompensated—results in motion artifacts. To circumvent that, different strategies can be used: (i) imaging in breath hold, (ii) prospective gating and (iii) retrospective gating (RG). In clinical CT, investigating compliant patients in combination with extremely short acquisition times allows for breath-hold imaging. Breath-hold imaging in preclinical studies with non-compliant subjects however requires intubation and ventilation, the latter interrupted during the acquisition process. Hence, preclinical breath-hold imaging is invasive and in mice does not permit acquisition times much longer than 30 s. Thus, multiple studies proposed dividing the acquisition process in several breath hold phases followed by resting times in which the subject is ventilated but the acquisition is paused^2^. This strategy does, of course, prolong the total acquisition for the animal and is solely based on the assumption that position and shape of the lung in each breath-hold phase is identical. In contrast to that, prospective gating uses an external trigger signal to stop the acquisition at unwanted expansion states of the chest. Since respiratory rates of mice are much faster than in humans, even under anesthesia, prospective gating of the CT acquisition process is technically challenging as it would require abrupt starting and stopping of the CT gantry and/or blockage of the X-ray tube with a fast enough shutter to avoid additional irradiation of the subject. Therefore, so called retrospective gating (RG) is typically employed in preclinical CT imaging^3^. RG is a technique in which more than the required set of angular projection images is recorded, typically by performing additional rotations. After the acquisition (retrospectively) the phase of the breathing motion is derived from the acquired data and the projections are sorted accordingly into different subsets. These subsets are then reconstructed independently mimicking CT imaging in a static situation. While RG effectively increases the quality of the reconstructed data and can be easily implemented, it requires significantly higher X-ray dose levels than non-gated CT imaging as typically only a fraction of the acquired data is used for the final reconstructions. RG has been successfully applied in various studies^4–6^ and based on that success it is implemented in virtually all commercially available in-vivo microCT scanners. If prospectively or retrospectively gated in-vivo microCT is a better choice in terms of the trade-off between obtained information and needed X-ray radiation dose is difficult to answer as virtually no comparison studies are published using a device optimized for both techniques. In clinical CT for instance ECG gating for CT based angiography^7^ found shorter total acquisition times in prospective gating and therefore low X-ray doses. Whereas comparison studies in preclinical imaging are sparse Blocker et al. demonstrated that RG improves delineation of sarcoma metastasis within mouse lungs but was still outperformed by prospective gating^8^. It needs to be mentioned that the RG reconstruction used less projection images and a different reconstruction algorithm resulting in a lower SNR compared to the prospective gated data. However, they state: ”these data suggest that retrospective gating helps alleviate some, but not all, of the motion-related issues with tumor volumetry with a shorter acquisition time than prospectively gated lung CT”, which would translate to a lower radiation dose.

In addition to the visualization of anatomical changes within the lung, the assessment of the functional aspects of breathing is of great interest. In general, plethysmography is used for pulmonary function testing as it does not require a compliant subject unlike spirometry, the method of choice to assess respiratory function in patients^9^. However, the application of plethysmography in mice is rather difficult and the interpretation of the measurement results is challenging due to commonly overlooked influences of temperature and humidity conditions^10^. In recent years we established a X-ray based lung function measurement (XLF) that employs cinematic planar low dose X-ray imaging to determine lung function parameters in mice^11^. We showed that the high sensitivity of XLF allowed monitoring lung function non-invasively over time in different mouse models of allergic airway inflammation^12,13^. Although XLF introduces a comparably low X-ray dose of approximately 6 mGy, it requires a further measurement in addition to lung CT imaging used to derive anatomical information, thereby prolonging the time in which the mouse needs to remain under anesthesia. Thus, we present a new approach to quantify respiratory motion in the raw data of low dose retrospective gated lung CT of free breathing anesthetized mice that circumvents the need for additional X-ray dose and scanning time by an otherwise subsequently performed XLF acquisition. Since, we are interested in quantifying the respiratory motion over the entire breathing cycle, prospective gating which only acquires data at particular time points cannot be applied. RG on the other hand has already been used for lung function measurements in combination with forced ventilation^14^. In that particular study phase contrast CT was used to reconstruct lung CT data sets at 16 time points throughout the entire respiratory cycle as a base to calculate regional airflow. However, in this study free propagation phase contrast CT was employed—a technique that is virtually only accessible at synchrotron sources. In contrast to that we present a more limited but simple approach.

To illustrate the potential of our novel retrospective gating based X-ray respiratory motion measurement approach (rgXLF) we performed rgXLF and regular planar XLF in the *mdx* mouse (C57BL/10ScSn-Dmdmdx/J (Bl10/mdx)) in comparison to healthy control mice. *Mdx* mice present the most common and well established mouse model used for studying Duchenne muscular dystrophy (DMD)^15^. DMD is characterised by progressive skeletal muscle degradation, gradual weakening of the diaphragm and auxiliary respiratory muscles^16^. In addition, the skeletal morphology of the thorax/spine is changed over the course of the disease. The patients present with reduction in respiratory function which is recapitulated in the *mdx* phenotype. Especially a reduction in the tidal volume of the mdx mice is frequently reported^17^. By assessing the *mdx* mouse model with rgXLF we succeeded to accurately reproduce the 3D anatomy of the lung and structure of the thoracic bone as well as to derive functional parameters of breathing motion comparable to standard XLF. We achieved that by analyzing also those projection images which in the original implementation of RG are simply discarded (Tables 1, 2).

**Table 1:** MicroCT acquisition parameters.

Tube voltage	90 kV
Tube current	100 $\upmu$A
Field of view (FOV)	20 $\times$ 20 mm$^2$
Pixel size	39 $\upmu$m
Total acquisition time	34 s
Rotation angle	720$^\circ$
Total amount of projection images	1028

**Table 2:** Parameter for X-ray based lung function measurement.

Tube voltage	90 kV
Tube current	40 $\upmu$A
Field of view (FOV)	20 $\times$ 20 mm$^2$
Pixel size	39 $\upmu$m
Total acquisition time	34 s
Rotation angle	0$^\circ$
Total amount of projection images	1024

## Results

Cinematic X-ray imaging has already successfully been used to quantify breathing motion. The breathing changes the expansion state and the air content of the lung, which in turn modulates the X-ray attenuation and can therefore be detected as a change in the intensity of the lung region over time^12^.

### Principle

**Figure 1:**
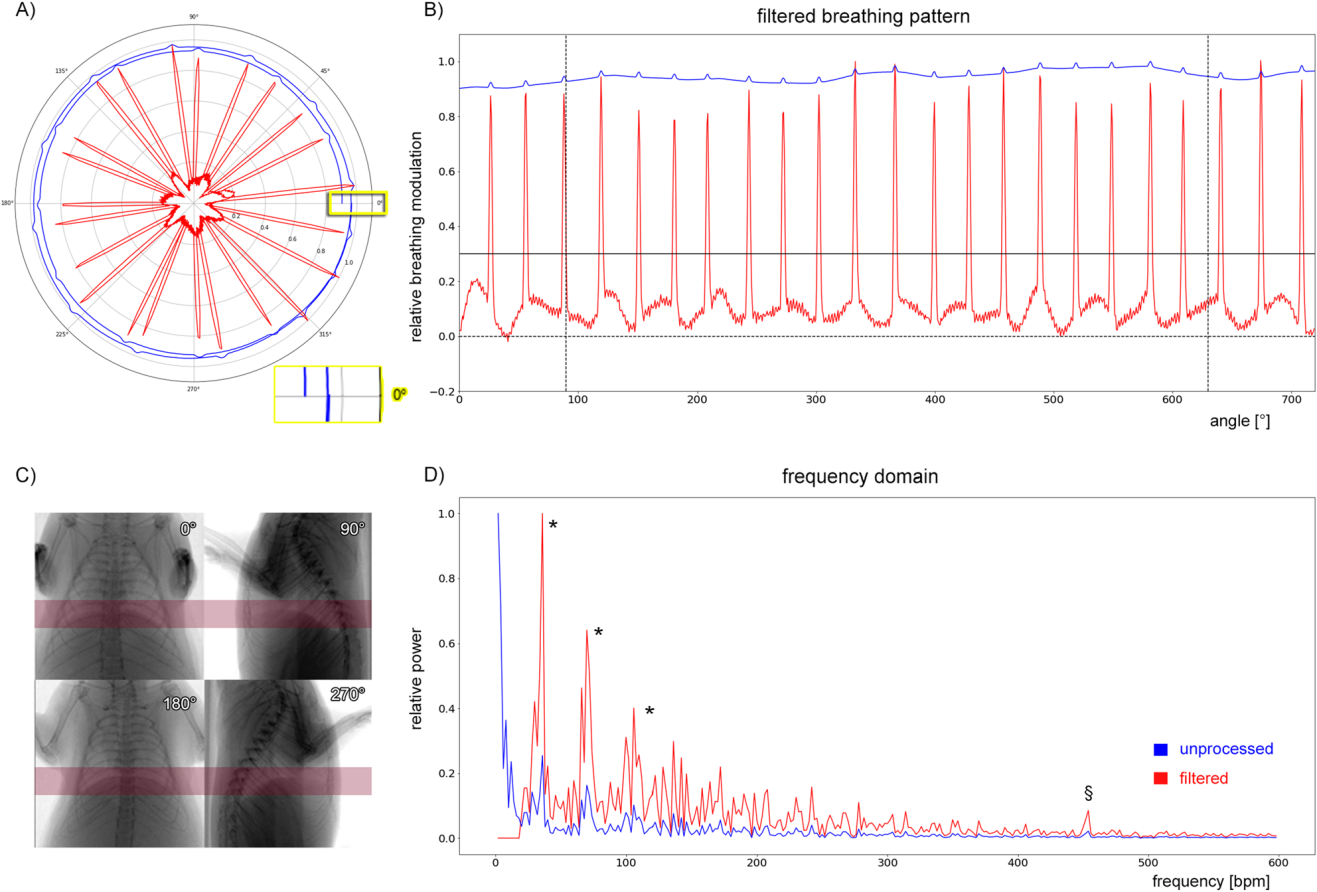
Principle of retrieving the breathing motion from the set of angular distributed projection images. (**A**) Polar plot of the average X-ray transmission over a region of interest (ROI) covering the entire cross-section of the mouse at the lung-diaphragm interface (blue) is shown. The breathing is only visible as subtle modulation of the signal and therefore a background correction is need, which is hindered by the fact that the signal is not strictly 2*pi* periodic (insert). (**B**) The final breathing pattern after background correction is shown in red. To suppress potential imprecision in the background correction and the beginning and end of the acquisition a range of 90$^\circ$ from the start and end of the measurement (vertical dashed lines) was discarded. The amplitude of the remaining angular range was normalized to [0,1]. A level of 0.3 (horizontal dashed line) was used to identify breathing events in the resulting function. (**C**) Representative projection images are shown at 0, 90, 180 and 270$^\circ$ with the ROI indicated in red. (**D**) Comparison of the power spectra of the unprocessed data (blue) and the processed data (red) shows that after background removal the breathing events and their harmonics can clearly be seen (* asterisks). Moreover, the frequency of the heart beat can be detected as well (§). *The figure was generated using matplotlib 3.5.1* www.matplotlib.org*and gimp 2.10.28* www.gimp.org.

To enable quantification of the respiratory motion this information needs to be extracted from the acquired projection images. To this end the average brightness in a user-defined rectangular region over the lung-diaphragm interface is analyzed as indicated in red in Fig. 1D. Breathing modulates the position of the diaphragm and the expansion state of the chest both resulting in a change in the average X-ray transmission over time $U(\alpha )$ (blue-curve, Fig. 1A). However, the angular projection of the mouse anatomy as well as the modulation of the X-ray tube intensity caused by the electronics contribute more strongly to the obtained X-ray attenuation function than the breathing as evidenced by the large baseline of obtained function (blue) in both the polar and linear plot Fig. 1A,B. To remove this unwanted effects we exploited the facts that the data should be periodic in 360$^\circ$ and that the anatomy of the mouse is mostly reflected in the first *K* frequencies of the Fourier transformation of the signal. However, the insert in Fig. 1A shows that the modulation of the intensity of the X-ray tube yielded in a non-2*pi*-periodic function. We therefore extended the function by adding its mirrored version, which then always results in a periodic function in 4*pi*. Thus, we reconstructed the background signal by inverse Fourier transformation of the first *K* frequencies and subtracted the results from the original data, resulting in the red trace Fig. 1A, which is also shown in a linear plot in Fig. 1B. (Note: for the demonstrated example $K = 20$ was used). It can be seen in Fig. 1A,B (red curves) that the breathing peaks are now well defined. To further suppress potential imprecision in the background correction at the beginning and end of the acquisition, the angular ranges from 0–90$^\circ$ to 630–720$^\circ$ are discarded as indicated by the black dashed vertical lines in Fig. 1B. The amplitude of the remaining angular range was scaled between 0 and 1 and a level of 0.3 was used to detect breathing events (horizontal black line in Fig. 1. Example projection images are illustrated in Fig. 1C at angles of 0, 90, 180 and 270$^\circ$. The normalized power spectra shown in Fig. 1D demonstrate that once the strong contributions of shape of the mouse (blue, beginning of the spectrum) is removed the breathing events and their harmonics (asterisks) can clearly be observed. Moreover, in the filtered spectrum (red) a peak at approximately 470 bpm is visible Fig. 1D (§) depicting the heart rate of the mouse Fig. 1D. This trace is used for both deriving functional parameter and sorting the projection images to perform RG CT reconstruction.

### X-ray dose measurements

A commercial X-ray dose measurement system was used to measure the dose length product for several acquisition protocols as summarized in Table 3. Wrapping the probe with a 1 cm layer of pork to mimic scattering processes in the mouse did not affect the readings substantially. Note, that the standard CT acquisition protocol of 17 s, includes ramping up the tube voltage for 2 s resulting in a total exposure time of 19 s. Therefore, our acquisition protocol for rgXLF using a tube voltage of 90 kV, a current of 100 $\upmu$A, a field-of-view (FOV) of 20x20 $\hbox {mm}^2$ and an acquisition time of 34 s results in a dose of approximately 37 mGy. Whereas the planar XLF measurement performed with the same parameters apart from the tube current of 40 $\upmu$A and a total acquisition time of 30 s, results in a total X-ray dose of approximately 13 mGy.

**Table 3:** Dose measurements for different settings.

Covered	Voltage [kV]	Current [$\upmu$A]	FOV [mm$^2$]	Pixel size [$\upmu$m]	Time [s]	Dose [mGy]
–	90	200	73 $\times$ 73	143	19	14.0
–	90	200	73 $\times$ 73	143	19	13.9
–	90	200	73 $\times$ 73	143	19	14.8
–	90	200	40 $\times$ 40	78	19	21.3
–	90	200	20 $\times$ 20	39	19	41.6
x	90	200	73 $\times$ 73	143	19	13.8
x	90	200	40 $\times$ 40	78	19	20.0

Measurements taken with wrapping the probe with a layer of 1cm meat to mimic scattering within a mouse are indicated by ’x’. Please note that a smaller FOV (smaller pixel size) is achieved by moving the X-ray source closer to the mouse/specimen which results in elevated dose levels.

### Parametrization of the breathing pattern

In order to parameterize the obtained breathing pattern we applied the same strategy described by Khan et al.^11^. In short, a level function at 30% of the relative X-ray attenuation signal was used to identify single breathing events. The start of the inspiration phase was defined as point with the highest curvature prior each breathing peak. The expiration phase is defined as as descending part of the curve from the peak till the start of the next breathing event is reached. Although, several parameters can be calculated for each individual event^11^ in this study we focused on the parameter *k* of a function $f(t) = I_{0}\exp (-k \times t^{2})+c$ fitted to the expiration phase and of the heart rate measured in Fourier space. Since, the underlying lung motion is more complex the used function presents a simplification. The magnitude of the movement of the lung tissue increases towards the position of the diaphragm, thus size and placement of the region affects the calculated k-value as shown in Supplemental Fig. 1A. Therefore, similar regions should be used for comparison of the bulk motion of the lung between different subjects. To increase the robustness fit and to account for the noise and limited temporal resolution of the data, the data of all measured expiration phases are overlaid (Supplemental Fig. 1B).

### Validation of the RG based respiratory motion measurements in the *mdx* mouse model

**Figure 2:**
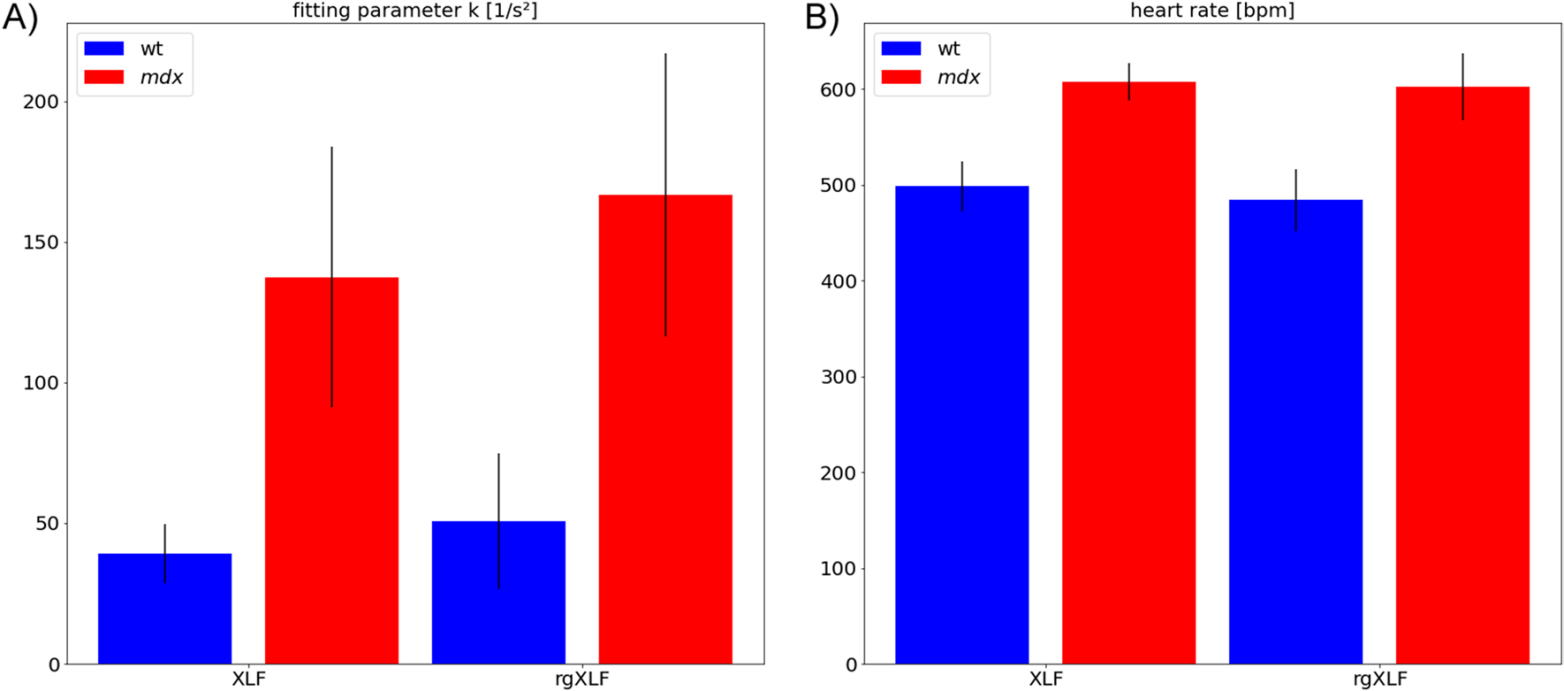
Comparison of the novel RG based lung function measurement (rgXLF) with the established planar XLF. (**A**) shows the calculated k-values for the expiration phase in *mdx* and wt mice, revealing increased k-values in *mdx* mice. (**B**) The calculated heart rates for *mdx* and wt mice demonstrate a faster rate for *mdx* mice than in wt mice at the same level of anesthesia. Both XLF and rgXLF show similar outcomes that correlate with a Pearson correlation coefficient of 0.92 in both parameters. (Error bars present standard deviation.)*The figure was generated using matplotlib 3.5.1* www.matplotlib.org*and gimp 2.10.28* www.gimp.org.

To evaluate the accuracy of rgXLF we compared it to the established planar XLF method performed subsequently in the same *mdx* mice and wild type controls (wt) applying the same analysis pipeline described above. Figure 2A,B demonstrates that in both methods the k-value of the expiration phase as well as the heart rate showed similar results (Pearson correlation coefficient of 0.92 for the k-value and heart rate). Moreover, both methods allowed to successfully discriminate *mdx* from wt mice (Mann-Whitney U = 0 for both k-value and heart rate) and revealed an elevated k-value and heart rate in *mdx*. Note, that XLF and rgXLF have been performed subsequently. Due to the variations of the breathing rates of 15% on average between the measurements a perfect correlation can not be expected.

### Retrospectively gated CT reconstruction

**Figure 3:**
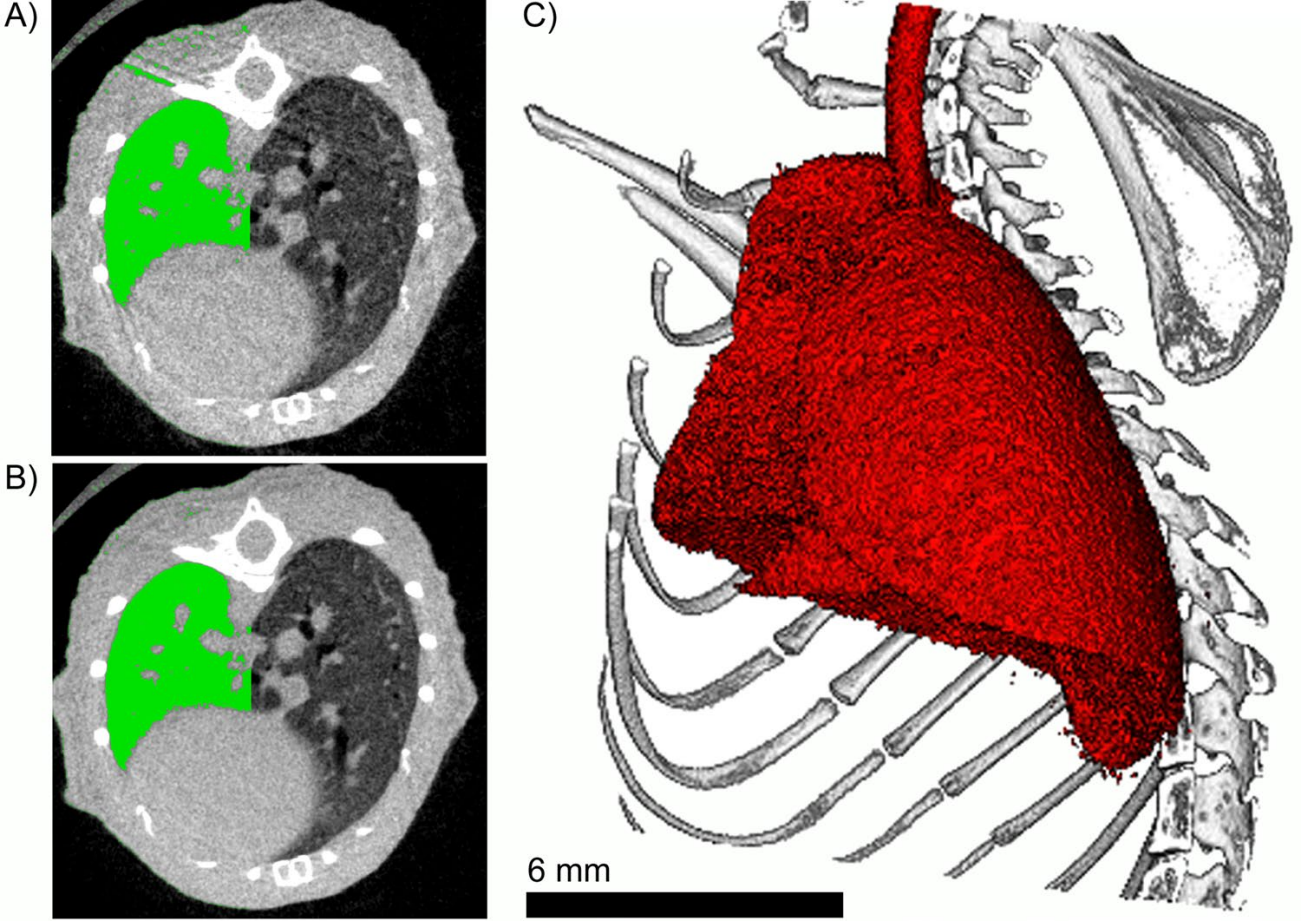
Representative cross section of CT data sets of the chest region of a mouse reconstructed (**A**) without RG and (**B**) with RG and frame averaging. In green the segmented lung using the same threshold is partially overlaid. Clearly, in (**B**) both, less motion artifacts and a sharper delineation of the lung towards the rib cage are observed. This allows to study the anatomical shape of the lung in 3D as demonstrated in (**C**). Note, that the interface between lung and heart was not improved since no gating was performed for the movement of the heart. *The figure was generated using imagej 1.53f* www.imagej.nih.gov/ij/, *gimp 2.10.28* www.gimp.org*and scry (proprietary 3D render software)*.

The data derived breathing curves (exemplary shown in Fig. 1B) were used to sort the angular projections acquired over 720$^\circ$ rotation into two bins: (i) inspiration and (ii) expiration. Since the inspiration phases are very short, only a few projections are recorded. Thus, only predominately the expiration phase was 3D reconstructed. To this end we used the following scheme. Since, each projection angle between 0 and 360$^\circ$ has been acquired twice we generate a new data set over 360$^\circ$ taking either the corresponding projection from the first or from the second rotation depending on which showed the lowest value in the calculated breathing curve. If both projections in the first and second rotation had a value below 0.1 the average of both frames was used. In addition, the amount of angles at which no frame was found to have a value of less then 0.3 was reported as a measure of the reliability of the approach. Since we applied a standard filtered back projection algorithm for 3D reconstruction, which requires a set of equally distributed angular projections, only in cases in which the breathing events do not largely overlap between the 1st and 2nd rotation, we achieved reconstructions with a sharper delineation of the lung and the absence of motion artifacts as shown in Fig. 3B in contrast to the reconstruction demonstrated in Fig. 3A obtained without applying RG. Averaging the two frames at the same angle, if both belong to the expiration phase, reduced the noise level and therefore further improved the image quality. This is helpful if the lung needs to be segmented for subsequent analysis as demonstrated in Fig. 3C.

### Combined functional and anatomical characterization of the *mdx* mouse model

**Figure 4:**
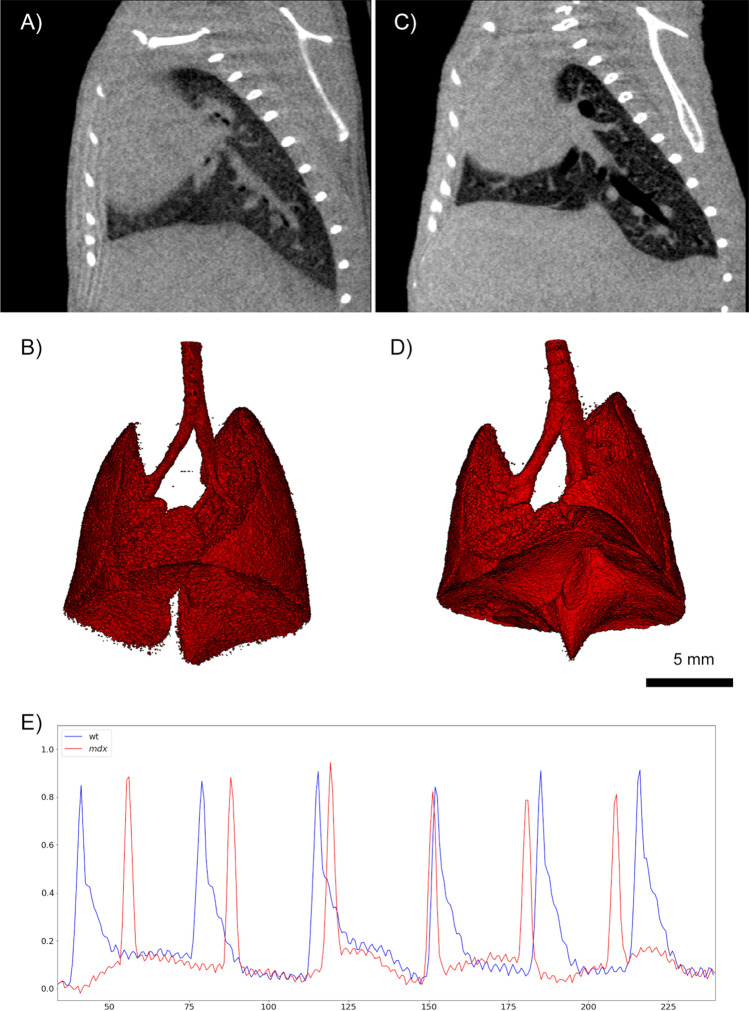
Cross sections of (**A**) a CT image of a healthy control in comparison to (**C**) of a *mdx* mouse show a modified shape and an altered position of the diaphragm in the *mdx* mouse. The same deformation of the lung can be observed in the 3D renderings of the segmented lung of the healthy mouse (**B**) and the *mdx* mouse (**D**), especially for the post-caval lobe (§). (**E**) shows the extracted breathing pattern for the same mice (healthy = blue and *mdx* = red). Clearly the *mdx* mouse displays a more rapid decay in the expiration phase.*The figure was generated using imagej 1.53f* www.imagej.nih.gov/ij/, *using matplotlib 3.5.1* www.matplotlib.org, *gimp 2.10.28* www.gimp.org*and scry (proprietary 3D render software)*.

The use of our improved RG approach allows to simultaneously quantify anatomical and functional differences in the *mdx* mice compared to their wild type controls. In Fig. 4 representative cross-sections, lungs segmented in 3D and parts of the isolated breathing events are shown for one wild type control Fig. 4A,B and a *mdx* mouse Fig. 4C,D. For segmenting the lung envelope a simple threshold based segmentation followed by manual removal of the air outside the mouse was used. As threshold the arithmetic average between the mean grey values of the lung region and soft-tissue was employed. Already the cross-sections Fig. 4A,C demonstrate that the shape and position of the diaphragm is dramatically different between the *mdx* and the control mouse. This is further demonstrated in the 3D renderings of the segmented lungs. In *mdx* post-caval lung lobe appears enlarged and elongated towards the abdomen. Additionally, the lumen of the airways is increased in the *mdx* animal (Fig. 4B,D). Figure 4E show the traces of the breathing events extracted from the raw-data sets of the CT acquisitions according to the principle described above. Clearly, a more rapid decay (larger k-value) was evidenced in *mdx* mice (red) compared to healthy controls (blue). In addition, the high frequency modulations of the traces represent the heart beat.

## Discussion

Here we present an extension of the known retrospective gating (RG) approach for *in* = /*vivo* lung CT imaging evaluated in a mouse model of Duchenne muscular dystrophy (DMD). We demonstrate that when careful filtering is applied, the average X-ray attenuation at the chest region can be used to quantify differences in the breathing motion. The comparison with our established planar X-ray based lung function measurement (XLF) showed that the same k-values of the expiration phase and similar heart rates were obtained. The application of both methods in a *mdx* mouse model revealed increased k-values and elevated heart rates under isoflurane anesthesia adjusted to a breathing rate of 0.7 Hz. Since a strong correlation between rgXLF and classical XLF was found, an additional imaging session to measure respiratory motion is no longer needed and the redundant data of RG can now be used more efficiently.

The original RG approach described by Bartling et al. is applied to suppress motion artifacts of the lung at the cost of elevated X-ray dose levels^3^. The breathing motion that gets detected in the acquired data set is used to re-bin the data. RG has been successfully applied in variety of CT imaging studies in commercial in-vivo microCT systems^4,5^ as well as using experimental setups at for instance synchrotron sources^14,18,19^ to only name a few. Bayat et al. demonstrated that retrospective gating can be used to perform 4D CT imaging in rats to study the movement of different lung areas in detail, to follow recruitment and local collapse of alveolar spaces and potentially in the future to derive maps of local elasticity^18^. Dubsky et al. showed for ventilated mice that RG in combination with phase contrast CT imaging can be used to even extract regional airflow^19^. However, most of those studies employ much more than 2 rotations to be able to successfully gate the data, resulting in elevated X-ray dose levels. Our imaging protocol applies much less X-ray dose but with a voxel size of 40 um it can not be used to resolve the lung structure of a mouse in detail. Thus, pathological changes can only be detected if they affect the lung on a larger scale. However, our protocol with a dose of approximately 37 mGy in combination with a short total acquisition time of only 34 s enables longitudinal experiments in free breathing anesthetized mice. In future, the derived lung function parameters have the potential to supplement other measurements such as whole body plethysmography and may on its own permit monitoring disease progression or therapy response in a simple way.

The here presented approach of rgXLF requires only to define an area crossing the lung. The movement of the lung differs at different locations as for instance shown by Ref.^14^. Especially, regions close to the diaphragm experience stronger movement. Thus, the obtained parameter depend on the size and placement of the region of interest as demonstrated in Supplemental Fig. 1. Therefore, the regions should be defined in a similar way in all measurements and due to the noisy nature of the low dose acquisition should be sufficiently large. Apart from that, no additional assumptions are made. Therefore, the method can also be applied in a local tomography scanning situation^20^, in which not all projection images show regions of free air around the subject. Moreover, the approach is generally applicable to any CT system, if the acquisition frame rate is sufficiently high to allow sampling of the breathing motion and access to the acquired raw data is given. In our case, an isoflurane anesthesia was adjusted to achieve a breathing frequency of 0.7 Hz, which in combination with the 30 Hz frame rate of the detector enabled to retrieve a significantly different k-value between *mdx* mice mimicking DMD and their healthy controls. The standardization of the breathing rate was done to ease the comparison between the different mice, to relate the results to previously performed experiments using the planar XLF approach^11–13^ and to respect the low frame rate of 30 fps of the detector. Adjusting the breathing rate was easier to perform than to precisely standardize the isoflurane concentration. However, the breathing rate at a given isoflurane concentration might present an important parameter of a lung disease which we in the moment cannot exploit.

In addition, adjusting the breathing rate allows to ensure that breathing events do not occur at the same angles of—in our case—an acquisition that consists of two consecutive rotations. The entire pipeline is integrated in our free available X-ray based lung function measurement software that can be downloaded at https://gitlab.com/heimdall32/xLFinal. However, the rgXLF approach has its limitation as the contributions of the underlying shape of the mouse can not completely be removed from the breathing signal. Thus, the rgXLF will most likely show limited sensitivity for features affecting tidal air volume, signs of inflammation or fibrosis etc. However, rgXLF as demonstrated here allows to reliably retrieve temporal parameters such as the k-value, relative inspiration time, heart rate and more. Due to the fact that standard RG always comes at the cost of elevated X-ray dose levels, rgXLF will be limited to preclinical research, but in this perspective allows addressing lung anatomy, shape of the thoracic bone and respiratory motion simultaneously. Thus, we believe that our approach will be a valuable add-on to preclinical lung CT imaging approaches which typically already apply standard RG.

The k-value as a constant of a Gaussian function fitted to the expiratory phase is predominately measuring the effect of elastic recoil of the lung in combination with an later onset of a dampening effect of the diaphragm^21^ and is therefore especially sensitive for characterizing the *mdx* mice known for fibrotization of the diaphragm^22^. Clinically the expiratory time constant (RCexp) is a known measure that combines effects of changes in compliance and resistance. It is possible that the here presented k-value based on lung motion could present an easy, accessible surrogate marker for the RCexp, which will be validated in future studies.

## Methods

### In-vivo microCT imaging and retrospective gating

In-vivo microCT imaging was performed using the QuantumFX device (PerkinElmer) operated with the settings in Table 1.

### X-ray based lung function measurement (XLF)

For validation of the here presented novel imaging method we performed our established X-ray lung function measurements (XLF) based on planar cinematic X-ray imaging. For this purpose the same in-vivo microCT system was utilized with the following parameters Table 2. The acquisition was performed directly after the CT acquisition.

### X-ray dose measurements

A commercial dose measurement system ”Diados” type 11003, SN 0173 (PTW) in combination with a CT adapter ”Diados CT adapter’, SN: 000607, (PTW). We used a vented cylindrical pencil chamber for dose length product measuremnents in clinical CT SN: 001031 (PTW) with a sensitive measurement length of 30cm.

### Reconstruction and segmentation of microCT data sets

A subset of the acquired angular projections resembling a full rotation of 360$^\circ$ was reconstructed using the proprietary reconstruction software of the QuantumFX in-vivo microCT system (Perkin Elmer). This resulted in volume data sets with a matrix of 512 $\times$ 512 $\times$ 512 voxel and an isotropic voxel size of 40 $\upmu$m. In this data lungs were segmented with a simple threshold based segmentation algorithm implemented in Scry v6.0.

### MDX mouse model

Mdx mice (C57BL/ 10ScSn mdx) used for breeding were kindly provided by Ralf Herrmann (University of Essen, Germany). *Mdx* mice (2 male, 2 female mice) and healthy controls [C57BL/ 10ScSn] (2 male, 2 female mice, from here on referred to as wt) were used for lung function assessment by XLF and CT imaging. All mice were of an age of 30 weeks at the time of data acquisition. The animals were anaesthetised with approximately 2–3% isoflurane, airflow of 1 L/min of 50:50 mix of air and oxygen for both measurement methods adjusting the breathing rate to about 1.4 s in between inspiration events. Adjusting the breathing rate was done to ease comparability of the obtained measurements. 1.4 s were chosen to match the results of the previously establish planar lung function measurement approach^11^ and to account for the comparable low frame rate of 30 fps of the X-ray detector. Mice were placed in supine position. Depending on how much time was needed to adjust the breathing rate the mice were under anesthesia for a maximum of 5 min.

### Ethical statement

All animal in-vivo procedures were performed in compliance with the guidelines of the European Directive (2010/63/EU) and the German animal ethics regulations and were approved by the local ethics office (Niedersaechsisches Landesamt für Verbraucherschutz und Lebensmittelsicherheit, LAVES, ethics approval 33.9-42502-04-18/2763). Furthermore, the study was carried out in compliance with the ARRIVE guidelines (https://arriveguidelines.org).

### Software and statistics

Lung function measurement was performed with the custom made software xLFinal (https://gitlab.com/heimdall32/xLFinal). Plots were generated with matplotlib (https://matplotlib.org). Segmentation and rendering of the lung was done in Scry 6.0 a custom made software by the author. For statistical analysis a Pearson correlation coefficient as well as the Mann–Whitney U was calculated using PAST^23^.

## Supplementary Information

Supplementary Information.

## Data Availability

The datasets generated and/or analysed during the current study are available in the open science framework (OSF) repository, DOI 10.17605/OSF.IO/3QPXZ. The generated analysis software can be found here https://gitlab.com/heimdall32/xLFinal.
