# Supplementary material for: Simultaneous assessment of lung morphology and respiratory motion in retrospectively gated in-vivo microCT of free breathing anesthetized mice
Source: Sci Rep. 2022 Aug 2;12:13299. doi: 10.1038/s41598-022-17335-4 (PMC9345384; doi:10.1038/s41598-022-17335-4)
Supplement: Supplementary file 1 — Supplementary Information. [file 41598_2022_17335_MOESM1_ESM.pdf]

Supplemental material - Simultaneous assessment of lung morphology and respiratory motion in retrospectively gated in-vivo microCT of free breathing anesthetized mice

In order to demonstrate the effect of the position and size of the defined analysis region onto the calculated k-values four different regions as indicated in supplemental Fig. 1A have been analyzed. Additionally the result are compared to the classical planar XLF that uses 2 lung shaped regions (white, supplemental Fig. 1A). The k-values are calculated based on a fit of a Gaussian function into the descending/exhalation part of the breathing curve. To increase the robustness of that approach the data of all the identified breathing events are overlaid, which in our case using a breathing rate of 0.7Hz and a total acquisition time of 34s typically results in approx. 20 breathing events (supplemental Fig. 1B). Supplemental Fig. 1C and D show the obtained breathing curves for one *wt* and one *mdx* mouse respectively. The larger region (green) below the arms shows a more stable amplitude of the peaks and less effects at the baseline and was therefore selected for the analysis. Supplemental Fig. 1D shows that the traces for this region are similar to the once obtained in planar XLF. However, in planar XLF the baseline is more homogeneous pointing to the fact that no all contributions of the shape mouse were removed form the rgXLF data. Supplemental Table 1 summarizes the obtained results for each group. There is a clear tendency for shorter k-values closer to the diaphragm. However, in every different measurement areas a striking difference between *wt* and *mdx* mice was observed.

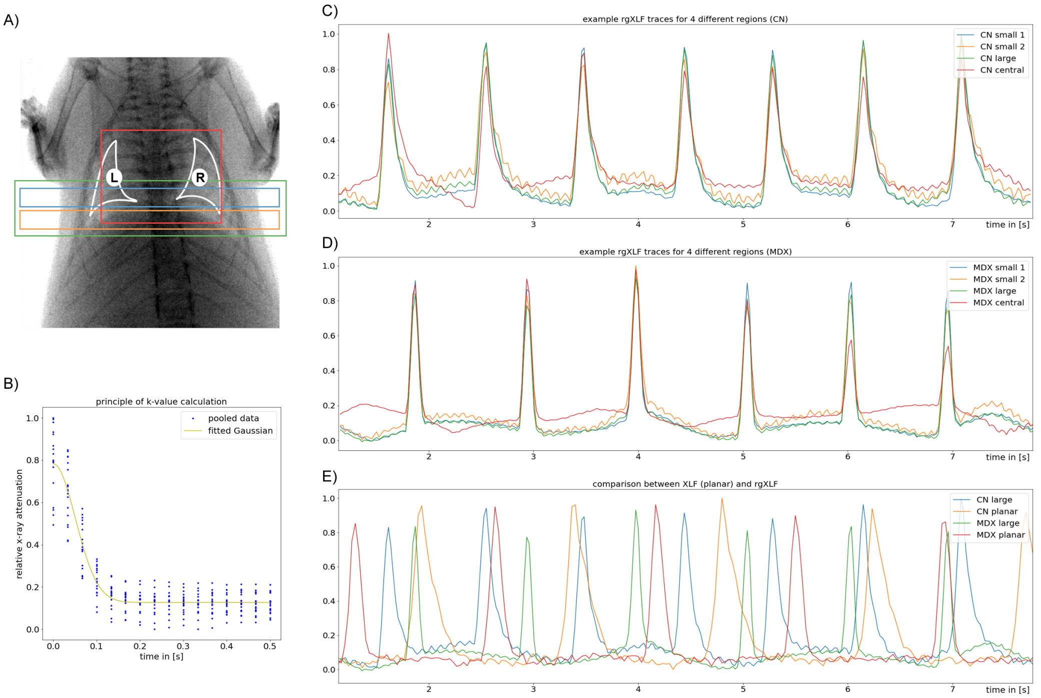

Supplemental Figure 1. A) shows the definition of different regions for evaluation of the dependence of the k-value calculation on that. The regions used for the planar XLF analysis (additional acquisition) are labeled in white. B) shows an example of the calculation of the k-value. A Gaussion function is fitted into the overlaid descending parts of all identified breathing events. C) shows the effect of selecting different regions onto the obtained traces in a control mouse. Since the central regions in addition also contains the projection of the arms background correction is more challenging resulting in compromised exhalation phases. D) shows the same analysis in an mdx mouse. E) shows overlaid the obtained traces of rgXLF and planar XLF measurements for one control and one mdx mouse. The traces appear pretty similar but show a homogenous baseline in the planar measurements.

The figure was generated using imagej 1.53f `imagej.nih.gov/ij`*, matplotlib 3.5.1* `matplotlib.org` *and gimp 2.10.28* `www.gimp.org`

region/method	wt	mdx
small 1	30.5±12.8	220.3±78.7
small 2	37.7±50.7	171.0±35.5
large	49.9±55.1	166.8±55.4
central	50.7±59.0	173.4±49.8
planar	27.6±9.1	127.0±47.5

Supplemental Table 1. shows the dependency of position of the region onto the measured k-value. Region small 1 is closest to the diaphragm and experience a more rapid movement resulting a smaller k-value. Despite that fact the differences between *wt* and *mdx* are maintained at all positions.
